# Detection and molecular characterisation of *Cryptosporidium* spp. in Swedish pigs

**DOI:** 10.1186/s13028-020-00537-z

**Published:** 2020-07-29

**Authors:** Emelie Pettersson, Harri Ahola, Jenny Frössling, Per Wallgren, Karin Troell

**Affiliations:** 1 Department of Animal Health and Antimicrobial Strategies, National Veterinary Institute, 751 89 Uppsala, Sweden; 2 Department of Clinical Sciences, The Swedish University of Agricultural Sciences, PO Box 7054, 750 07 Uppsala, Sweden; 3 Department of Microbiology, National Veterinary Institute, 751 89 Uppsala, Sweden; 4 Department of Disease Control and Epidemiology, National Veterinary Institute, 751 89 Uppsala, Sweden

**Keywords:** 18S rRNA, 28s rRNA, Cryptosporidiosis, *C. parvum*, *C. scrofarum*, *C. suis*, Genotyping, Parasite, Zoonosis

## Abstract

**Background:**

*Cryptosporidium* is a genus of apicomplexan parasites that cause enteric disease in vertebrates. In pigs, infections are most often asymptomatic, but may result in diarrhoea and poor growth. The most common species detected in pigs are *C. suis* and *C. scrofarum* with low zoonotic potential. *C. parvum*, with higher zoonotic potential, may also be found. As previous knowledge on the occurrence of *Cryptosporidium* in Swedish pigs is scarce, this was investigated in our study. Faecal samples from 13 pig herds were collected and a total of 222 pooled pen samples, from suckling piglets (n = 48), growers, aged 6–12 weeks (n = 57), fatteners, aged 13–24 weeks (n = 67) and adult animals (n = 50) were included. Samples were analysed using microscopy and positive samples were further analysed using polymerase chain reaction and sequencing of the 18S rRNA gene and the 28S rRNA gene to determine species.

**Results:**

*Cryptosporidium* spp. were detected in all sampled herds and in 25% (56/222) of the individual pen samples. Infections were most common in growers and fatteners with 51% (29/57) and 35% (20/67) positive samples in each group, respectively. The piglets had 8% (4/48) positive samples and adults had 6% (3/50). Species determination showed *C. suis* and *C. scrofarum* in piglets and growers, *C. scrofarum* in the fatteners, and *C. suis* and *C. parvum* in the adults. Although no mixed infections could be confirmed we saw signs of double peaks in the 28S rRNA gene chromatograms, possibly indicating more than one species present per sample.

**Conclusion:**

*Cryptosporidium* spp. were detected on every sampled farm and in 25% of the individual pen samples in our study. We therefore conclude that *Cryptosporidium* spp. are present and likely common in Swedish pig herds, where pigs are loose and reared on solid floors. However, none of the farms reported any problems with poor weight gain, diarrhoea, or reduced appetite in their pig herds. The pig adapted *C. suis* and *C. scrofarum* were the predominant species identified. Two samples were positive for the more zoonotic *C. parvum*, and pigs should hence not be disregarded as a possible source of zoonotic cryptosporidiosis.

## Background

*Cryptosporidium* is a genus of apicomplexan parasites that is globally spread and is composed of many different species and genotypes. *Cryptosporidium* can infect all vertebrates but many of the species are adapted to only one or a few hosts, such as the pig [1, 2]. Transmission is predominately faeco-oral and may be direct or indirect through the ingestion of contaminated food or water. Infection may cause enteric disease in both humans and animals, and cryptosporidiosis is considered an important zoonotic and food-borne disease [3]. *Cryptosporidium* was first reported in pigs in 1977 and two species, *C. suis a*nd *C. scrofarum* are known to be adapted to the porcine host. Other species, such as e.g. the zoonotically important *C. parvum*, have also been found in pigs [1, 4–7].

*Cryptosporidium* spp. are parasites with a direct life cycle and pigs become infected when they ingest infective oocysts from their environment. The infective dose of *Cryptosporidium* is as low as ten oocysts, and since such oocysts may survive well in the environment, the potential for spread to new hosts is high [8]. When ingested, the oocysts excyst in the small intestine of the pig and release sporozoites that invade the epithelial cells. The resulting damage to the intestinal lining, as well as a prostaglandin induced response, may result in a combination of a malabsorptive and secretory diarrhoea [9]. Subsequently, clinical signs in pigs may include diarrhoea, anorexia and poor weight gain [8, 10]. Subclinical disease is however common, and the degree of clinical signs appears to be species or genotype associated [4, 5, 11].

The global prevalence of *Cryptosporidium* in pigs varies, and is reported from 1 to 100% [4]. Previous knowledge of the occurrence of *Cryptosporidium* spp., as well as which species that are present in Swedish pigs is scarce.

In Sweden there are approximately 1300 registered pig producers and 2.6 million pigs are slaughtered annually. Around 2% of the pig farms are registered as organic and 2% as specific pathogen free (SPF) [12]. Fattening pigs are generally a three-breed cross, with dams being a cross between Landrace and Yorkshire and inseminated with either Hampshire or Duroc semen. With regards to *Cryptosporidium*, Sweden may be of particular interest as pigs by national law must be kept loose at all times and a minimum of 70% of the floor must be solid [13]. Dry sows are mostly kept in groups on deep litter straw beds and piglets are weaned at a minimum of 28 day of age. This type of more animal welfare friendly housing is different from most other European countries where pigs often are kept on fully slatted floors, without bedding material and were sows are fixated in crates for periods of time. The Swedish way of housing pigs may increase the risk of faecal contact and infection with *Cryptosporidium*, as well as increase the chance of parasite survival in the environment. The aim of this study was therefore to investigate the occurrence of *Cryptosporidium* spp. in Swedish pig as well as to determine what *Cryptosporidium* species were present, using molecular methods.

## Methods

### Selection of herds

Thirteen pig farms, located in the Mälaren valley and the provinces of Skåne and Småland, were selected. This geographical area is where most Swedish pig farms are located. The farms had between 50 and 400 sows and were selected by convenience as they were visited for other study purposes. Twelve of the farms housed the pigs indoors in pens, with either straw, peat, or wood shavings as litter material. One farm was organic where pigs in all age categories had access to outdoor paddocks. All herds included in this study practiced age segregated production from birth, where a group of sows enter a previously emptied and cleaned farrowing unit and the offspring are reared to market weight without mixing with pigs of other age groups. Pigs in Sweden are declared free from diseases on the former list A of the World Organisation of Animal Health (OIE), as well as from porcine respiratory and reproduction syndrome [14], Aujeszky’s disease [15] and atrophic rhinitis [16]. Surveillance also show that Swedish pig farms are free from salmonella [17, 18]. In Sweden, growth promoters have been banned since 1986, and the routine use of anti-protozoal agents or metaphylactic antibiotics is not carried out. One of the indoor farms included in the study was a specific pathogen free (SPF) farm, also declared free of *Mycoplasma hyopneumoniae*, *Actinobacillus pleuropneumoniae, Brachyspira hyodysenteriae*, swine influenza and sarcoptic mange [19]. SPF farms have high biosecurity and rarely introduce new animals into their farms.

### Sample collection

Faecal samples were collected during the period of October 2017 until October 2018. A total number of 222 samples, from the 13 farms, were collected from pigs in the categories (i) piglets, 0–5 weeks (n = 48); (ii) growers, 6–12 weeks (n = 57); (iii) fatteners, 13–24 weeks (n = 67); and (iv) adult animals, older than 6 months (n = 50) (Table 1). All samples were pooled pen samples, collected from the floor of individual pens. Each sample represented one pen and no pen was sampled more than once. Each pen housed approximately 10 to 15 animals. Only fresh samples were collected, and care was taken to include several faecal piles into one pooled sample. Faecal samples varied in consistency from firm to soft, but no samples were loose or diarrhoeic. As samples were collected from partly slatted floors or deep litter beds, diarrhoeic samples could however have been missed. The samples were collected in individual plastic bags and kept cool during transport and storage until analysis was carried out. Faecal examination with microscopy was done within 1 week of receiving the sample. No clinical examinations were performed of the pigs. However, none of the farms reported any problems with poor weight gain, diarrhoea, or reduced appetite in their pig herds.

**Table 1:** Summary of the sampled Swedish pig herds included in the study

Farm no	Housing system	Production system	Total no. of samples	Piglets	Growers	Fatteners	Adults
1	Indoor	Farrow to finish	10	–	5	5	–
2	Indoor	Farrow to finish	16	5	5	5	1
3	Indoor	Farrow to finish	9	2	1	1	5
4	Indoor	Farrow to finish	19	4	5	5	5
5	Indoor	Farrow to finish	18	3	5	5	5
6	Indoor	Farrow to finish	20	5	5	5	5
7	Indoor	Farrow to finish	19	4	5	5	5
8	Indoor	Farrow to finish	21	3	6	6	6
9	Indoor	Farrow to finish	20	5	5	5	5
10	Indoor	Fattening farm	15	–	–	15	–
11	Indoor	Piglet producer	20	10	5	–	5
12	SPF	Farrow to finish	20	5	5	5	5
13	Organic, outdoor	Farrow to finish	15	2	5	5	3
	Total		222	48	57	67	50

The table shows a summary of the sampled herds included in the study, including the housing systems, types of production and the number of samples from each age category that was sampled

*SPF* specific pathogen free

### Faecal examination

Oocysts were isolated in a similar way to what has been described by Maddox-Hyttel et al. [10]. In brief, samples were initially prepared by suspending 1 g of faces in 7 mL of phosphated buffered saline (PBS) with Tween 20 (PBS-Tween). Care was taken to ensure that the 1 g of faeces was collected from different parts of the pooled sample. The suspension was filtered through a fine mesh sieve and underlaid with a saline-glucose flotation solution (glucose with saturated saline 50 g/100 mL, diluted 1:1 with MilliQ water, final specific gravity = 1.07 g/mL) to a total volume of 12 mL and centrifuged at 100×*g* for 3 min. The supernatant was transferred to a clean tube, washed 3-4 times using MilliQ water and centrifuged at 1400×*g* for 10 min. A sample volume of 1 mL was finally obtained, and 10 μL of each cleaned sample were placed on a Teflon printed 3-well slide (Immuno-Cell Int, Belgium) and air dried for > 30 min before fixation with acetone for 5 min. When dried, the wells were stained with diluted (1:20 with a buffer solution) fluorescein isothiocyanate (FITC)-labelled monoclonal anti-*Cryptosporidium* antibodies (Waterborne Inc., New Orleans, LA, USA) according to the manufacturer’s instructions. The wells were then examined using epifluorescence microscopy and oocysts quantified at 250× magnification and expressed as oocysts per gram faeces (OPG). The theoretical lower detection limit of this method was 100 OPG.

### Purification, amplification, and sequencing

DNA was isolated from positive samples using DNeasy PowerLyzer PowerSoil Kit (50) Ref. 12855. One millilitre of saline-glucose floated faecal suspension was briefly vortexed and transferred to 2 mL capped Eppendorf tubes. The tubes were centrifuged for 3 min at 14,500×*g*. The supernatant was removed and 750 µL of MoBio bead solution was added. The pellet was resuspended by vortexing, transferred to tubes containing the beads, where after 60 µL of solution C1 was added. After a brief vortex, the samples were incubated at 100 °C for 10 min. The manufacturers purification protocol was followed from the bead-beating step until elution, which was done in 80 µL of solution C6.

Purified DNA from the samples was amplified using a nested polymerase chain reaction (PCR). The primers used for the 18S rRNA gene nested PCR were according to Xiao et al. [20]. The reactions were run in a total volume of 25 µL and consisted of 5 µL 5× buffer A, 1.25 µL of both primers (stock 10 µM), 0.25 µL dNTP (stock 20 mM), 0.1 µL KAPA 2G Taq polymerase (KR0380, Kapa Biosystems), 2 µL of template and H_2_O up to 25 µL. The reaction conditions for the first round were initial denaturation at 95 °C for 3 min followed by 40 cycles at 95 °C for 30 s, 61 °C for 30 s and 72 °C for 30 s. A 2 min extension step at 72 °C completed the program. Two µL from the first reaction was used as template in the second PCR. The reaction conditions were the same as above except that the annealing temperature was raised to 63 °C.

In addition, seven samples, three identified as *C. suis*, three as *C. scrofarum* and one with no results from the sequencing of 18S rRNA gene, were used to determine a partial sequence of the 28S rRNA gene. The primers used for the 28S rRNA gene nested PCR were according to Koehler et al. [21]. The reactions were run in a total volume of 25 µL and consisted of 2.5 µL 10× buffer, 1.5 µL MgCl_2_ (stock 50 mM), 0.5 µL of both primers (stock 10 µM), 0.25 µL dNTP (stock 20 mM), 1.25 µL BSA (stock 5 mg/mL), 0.2 µL Platinum Taq polymerase (Invitrogen), 2 µL of template and H_2_O up to 25 µL. The reaction conditions for the first round were initial denaturation at 94 °C for five min followed by 35 cycles at 94 °C for 30 s, 58 °C for 30 s and 72 °C for 50 s. A five min extension step at 72 °C completed the program. Two µL from the first reaction was used as template in the second PCR. The reaction conditions were the same as above except that the extension was 30 s at 72 °C in each cycle.

The PCR fragments for sequencing were treated with ExoSap, sequencing reactions done with the inner primers using Big Dye Terminator v3.1 and the purified reaction run in an ABI 3500 genetic analyser. The obtained sequences were assembled using BioEdit 7.2.5 (http://www.mbio.ncsu.edu/BioEdit/bioedit.html) and compared with sequences in GenBank.

All unique sequences generated in this study have been deposited in GenBank under the accession numbers MN715855–MN715857 and MN718854–MN718857.

### Statistical analysis

Potential differences in occurrence of *Cryptosporidium* spp. across categories of pigs were investigated by examining descriptive statistics and applying Fisher’s exact test. P values were considered significant if < 0.05. Data management and statistical analysis was performed using Stata (StataCorp. 2017. Stata Statistical Software: Release 15.1. College Station, TX: StataCorp LLC.).

## Results

### Overall occurrence of *Cryptosporidium* spp.

*Cryptosporidium* spp. were demonstrated in all herds and in 56/222 (25%) of the total number of samples. The number of positive samples ranged from one to eight per herd, with a mean of 4.3 (SD ± 1.8). PCR and sequence analysis were able to identify the *Cryptosporidium* spp. in 55/56 *Cryptosporidium* positive samples but unable to clearly determine the species in one sample, which is referred to as unknown. Six farms had more than one *Cryptosporidium* spp. present at the farm level.

### Occurrence by age category

**Fig. 1:**
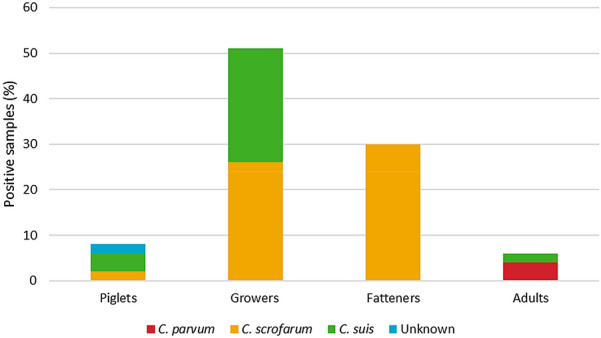
*Cryptosporidium* spp. identified in the different age categories of Swedish pigs sampled in the study. The figure shows the occurrence of *Cryptosporidium* spp. in each age category of the sampled pigs. It also shows what species of *Cryptosporidium* that were identified using PCR and sequencing of the 18S rRNA gene and the 28S rRNA gene. Piglets 0–5 weeks old (n = 48), growers 6–12 weeks old (n = 57), fatteners 13–24 weeks old (n = 67) and adults (n = 50). Species determination was not possible for one sample retrieved from piglets and is referred to as unknown

In suckling piglets, aged 0–5 weeks, *Cryptosporidium* spp. were detected in 4/48 (8%) of the samples with oocyst counts of 200 to 19,600 OPG. Species determination showed *C. suis* in two of the samples and *C. scrofarum* in one sample. In one sample, with an OPG of 300, species determination was not possible (Fig. 1). In growers, aged 6–12 weeks, 29/57 (51%) of the samples were positive with oocysts ranging from 100 to 30,600 OPG. Both *C. suis* and *C. scrofarum* were detected in this age category. In the fatteners, aged 13-24 weeks, 20/67 (35%) of the samples were positive with oocysts in the range of 100 to 5200 OPG and *C. scrofarum* was the only species detected. Finally, 3/50 (6%) of the samples from adult animals, older than 6 months of age, were positive, each sample with an oocyst count of 100 OPG. Species determination showed *C. suis* in one sample and *C. parvum* in two samples. There was no statistical difference regarding the occurrence and species distribution between the farms. However, regarding *C. suis* and *C. scrofarum*, there was a significant difference in species composition between the different age groups (P < 0.01). *C. scrofarum* was found more frequently in fatteners and *C. suis* was found in the younger grower pigs, as well as in one sow (Fig. 1).

No sequence of the *C. scrofarum* 28S rRNA gene was available in GenBank but the three samples identified as *C. scrofarum* showed two sequences variants in the present study (two with sequence MN718856 and one with MN718857) differing on three positions from each other. The 28S rRNA gene sequencing of the *C. suis* samples were confirmed in two of the samples although our sequences differed slightly from a published sequence in GenBank (Accession number KY882326). One sequence had five differences and a 3 base pair insert (MN718854). The other sequence had two differences and a 3 base pair insert (MN718855). In addition, there were several double peaks in the chromatograms implying more differences in this region. The third sample, identified as *C. suis* in the 18S rRNA gene, generated a *C. scrofarum* in the 28S rRNA gene sequence (identical to MN718856) indicating a potential double infection. This sample was collected from a pen of growers at an indoor farrow to finish farm. One additional sample, from a pen of piglets with 300 OPG, could still not be determined.

## Discussion

*Cryptosporidium* spp. were detected in all (100%) of the examined herds and in 25% of the total number of samples. Although the number of samples included in our study was relatively small (222), the high prevalence in the sampled group allowed us to conclude that this likely is a common parasite in Swedish pig herds. There are no previous data available on cryptosporidiosis in Swedish pigs but a study from Norway has reported 31% positive herds and 8.3% positive litters [22] which is low compared to our findings. In Denmark a herd prevalence of 16% in sows, 31% in piglets and 100% in weaners has been reported [10]. A more recent study in Denmark investigating the presence of *Cryptosporidium* in organic, outdoor pig herds found that 40.9% of the sampled pigs were positive [5]. Although the results from these Nordic studies cannot be directly compared to ours, due to the differences in study design and the larger number of sampled herds, they gave us an idea of the porcine *Cryptosporidium* prevalence in countries neighbouring Sweden.

Pigs of all ages may be infected by *Cryptosporidium*, as was seen in our study, although infections appear to be more common in younger animals such as growers and fatteners [1]. This is also the case for other animal species such as cattle [3, 10]. In this study we found the highest occurrence of *Cryptosporidium* spp. in growers aged 6–12 weeks and the lowest occurrence in the adults. These findings are similar to what has been reported from other countries such as Norway [22], Canada [8], Australia [2] and Denmark [10]. Cryptosporidiosis is most often subclinical in pigs but monoinfections as well as co-infections with for example *Cystoisospora suis* or rotavirus may induce clinical signs including poor growth, diarrhoea or even death [11, 23]. None of the sampled pigs in this study had noticeable diarrhoea or were reported to be in poor condition but clinical disease could not be fully ruled out as no clinical examination of the sampled animals was carried out, and diarrhoeic faecal samples could have been missed during sample collection. Possible co-infections were also not investigated.

In our study we used a combination of fluorescence microscopy and molecular diagnostics when analysing our samples. Microscopy was used to detect positive samples, and PCR and sequencing for species determination [24]. Using fluorescence microscopy allowed us to quantify the infection, something that is not possible when using PCR and sequencing alone.

C*ryptosporidium* oocysts may be shed from the host intermittently, and the chances of detecting positive animals may be higher if pooled samples are collected, as was done in our study [25]. None of the examined samples in our study were diarrhoeic and many studies have shown that there appears to be no association between the shedding of oocysts and diarrhoea in pigs [8, 10, 11], although the opposite has also been reported [22]. Out of the two *Cryptosporidium* spp. that are adapted to pigs, *C. scrofarum* and *C. suis,* the latter can be found in all age categories but more frequently during the pre-weaning period and prevalence of this species has also been shown to decrease with the age of the pig [4]. *C. scrofarum* on the other hand, has been reported to only infect pigs older than five weeks [4, 26]. This was further demonstrated when it was not possible to identify *C. scrofarum* in piglets experimentally infected with this species at the age of four weeks, but oocyst shedding did indeed occur in piglets aged 5 to 8 weeks [26]. *C. scrofarum* has however been reported in a few cases in pigs younger than 5 weeks, in for example China [27, 28]. In our study we found an age distribution of *C. suis* and *C. scrofarum* in accordance with previously published studies but did nevertheless isolate *C. scrofarum* from one sample collected in a pen of piglets aged 0–5 weeks.

Several species of *Cryptosporidium* have high zoonotic potential, including *C. parvum*. Pigs infected with this species may be asymptomatic or have clinical disease [4]. In our study only two samples were positive for *C. parvum* and both were collected from pens of adult sows, showing no clinical signs of illness, at two different farms. Mixed infections, with pigs harbouring more than one species of *Cryptosporidium*, have been reported [4], but in our study we were not able to confirm more than one species in each positive sample. However, one sample was identified as *C. suis* in the 18S rRNA gene while the sequencing of 28S rRNA gene showed a *C. scrofarum* sequence. In both cases the sequence was pure, and no underlying sequence was seen in the chromatograms. This could be due to a mixed infection and that the different PCRs are more prone to pick up one species over the other. This could never be confirmed as this was the only case of a potential mix in a single sample in our study, although four of the farms included had both *C. suis* and *C. scrofarum* present, and two farms had all three of the detected species present in the herd.

Both *C. suis* and *C. scrofarum* were detected in samples from the SPF farm that was included in the study. This herd was established by caesarean sections in 1988. No other animals have ever been introduced into this herd, and there are strict biosecurity measures on the farm. We can only speculate where the infection may have come from, but possibilities include transmission via fomites such as stable personnel or for example via rodents. Rats and mice have been found to carry the pig adapted species of *Cryptosporidium* [29] although none have yet been reported in rodents caught on Swedish pig farms [30].

Management of clinical cryptosporidiosis in pigs is supportive as there is no readily available therapeutic treatment or prophylaxis [3, 31]. Implementing good management and biosecurity systems, to ensure sufficient sanitation and to prevent environmental contamination of oocysts should instead be the focus [31, 32]. For example, solid floors with porous concrete have been associated with a higher prevalence of *Cryptosporidium* in pig herds compared to slatted floors [10, 32]. The stringent animal welfare laws in Sweden allows a maximum of 30% of the floor to be slatted and the large proportion of solid floors may contribute to the high occurrence of *Cryptosporidium* spp. in our study. In a study by Němejc et al. [4], the use of straw in the pig pens was shown to be strongly associated with cryptosporidiosis in the pigs. However, a previous study reported contradicting results, and a preventative effect by the use of straw was seen on the oocyst excretion [10]. All pigs in Sweden must have access to some sort of bedding material in the pens, and straw was most frequently used in the herds sampled in this study. Therefore, we had no access to animals raised without straw for comparison. *Cryptosporidium* has also been found to be more prevalent in pigs reared outdoors compared to indoors [5, 33]. Only one organic outdoor herd was included in our study and although no conclusion can be drawn from such small sample size, this was the farm with the lowest within-farm occurrence of *Cryptosporidium* spp. with only 1/15 (7%) positive sample, where *C. suis* was detected from a grower.

The most frequent species of *Cryptosporidium* found in this study were the pig specific *C. suis* and *C. scrofarum* that may affect growth rates and may cause clinical disease, especially if co-infections with other gastrointestinal pathogens exist. Zoonotic spread of *C. suis* and *C. scrofarum* is rare but has been reported [2, 34, 35]. In our study *C. parvum*, with a higher zoonotic potential compared to the pig adapted species, was detected in two samples from apparently healthy sows, but not from any growing pigs.

## Conclusion

*Cryptosporidium* spp. were detected on every sampled farm and in 25% of the individual pen samples in our study. We therefore conclude that *Cryptosporidium* spp. are present and likely common in Swedish pig herds, where pigs are loose and reared on solid floors. No clinical disease was however reported on any of the studied farms. The pig adapted *C. suis* and *C. scrofarum* were the predominant species identified. Two samples were positive for the more zoonotic *C. parvum*, and pigs should hence not be disregarded as a possible source of zoonotic cryptosporidiosis.

## Data Availability

The datasets used and/or analysed during the current study are available from the corresponding author on reasonable request.

## Abbreviations

*C. parvum*: *Cryptosporidium parvum*
*C. scrofarum*: *Cryptosporidium scrofarum*
*C. suis*: *Cryptosporidium suis*
FITC: fluorescein isothiocyanate
OIE: World Organisation of Animal Health
OPG: oocysts per gram
PBS: Phosphated Buffered Saline
PCR: polymerase chain reaction
SD: standard deviation
SPF: specific pathogen free
Tween 20: Phosphated Buffered Saline Tween
18S rRNA: 18S ribosomal ribonucleic acid
28S rRNA: 28S ribosomal ribonucleic acid

## References

[1] Kváč M, Kestřánová M, Pinková M, Květoňová D, Kalinová J, Wagnerová P (2013). *Cryptosporidium scrofarum* n. sp. (Apicomplexa: Cryptosporidiidae) in domestic pigs (*Sus scrofa*). Vet Parasitol.

[2] Johnson J, Buddle R, Reid S, Armson A, Ryan U (2008). Prevalence of *Cryptosporidium* genotypes in pre and post-weaned pigs in Australia. Exp Parasitol.

[3] Ryan U, Fayer R, Xiao L (2014). *Cryptosporidium* species in humans and animals: current understanding and research needs. Parasitology.

[4] Němejc K, Sak B, Květoňová D, Kernerová N, Rost M, Cama V (2013). Occurrence of *Cryptosporidium suis* and *Cryptosporidium scrofarum* on commercial swine farms in the Czech Republic and its associations with age and husbandry practices. Parasitol Res.

[5] Petersen H, Jianmin W, Katakam K, Mejer H, Thamsborg S, Dalsgaard A (2015). *Cryptosporidium* and *Giardia* in Danish organic pig farms: seasonal and age-related variation in prevalence, infection intensity and species/genotypes. Vet Parasitol.

[6] Zintl A, Neville D, Maguire D, Fanning S, Mulcahy G, Smith H (2007). Prevalence of *Cryptosporidium* species in intensively farmed pigs in Ireland. Parasitology.

[7] Kváč M, Sak B, Hanzlíková D, Kotilová J, Květoňová D (2009). Molecular characterization of *Cryptosporidium* isolates from pigs at slaughterhouses in South Bohemia, Czech Republic. Parasitol Res..

[8] Guselle N, Appelbee A, Olson M (2003). Biology of *Cryptosporidium parvum* in pigs: from weaning to market. Vet Parasitol.

[9] Fayer R, Xiao L (2008). *Cryptosporidium* and Cryptosporidiosis.

[10] Maddox-Hyttel C, Langkjær R, Enemark H, Vigre H (2006). *Cryptosporidium* and *Giardia* in different age groups of Danish cattle and pigs—Occurrence and management associated risk factors. Vet Parasitol.

[11] Schubnell F, von Ah S, Graage R, Sydler T, Sidler X, Hadorn D (2016). Occurrence, clinical involvement and zoonotic potential of endoparasites infecting Swiss pigs. Parasitol Int.

[12] Lannhard Öberg, Å., Marknadsrapport GRIS - utvecklingen till och med 2018. Rapport/Livsmedelskedjan och exportenheten. Jönköping: Jordbruksverket. Swedish Board of Agriculture. 2019.

[13] Jordbruksverket, Statens jordbruksverks föreskrifter och allmänna råd om grishållning inom lantbruket m.m. (SJVFS 2019:20) Jönköping: Jordbruksverket, https://lagen.nu/sjvfs/2019:20.

[14] Carlsson U, Wallgren P, Renström L, Lindberg A, Eriksson H, Thorén P (2009). Emergence of porcine reproductive and respiratory syndrome in Sweden: detection, response and eradication. Transbound Emerg Dis.

[15] Robertsson J. Status of the national eradication program of Aujeszky’s Disease. In: Swedish veterinary congress proceedings. Uppsala, Sweden. 1996.

[16] Wierup M, Wallgren P. Results of an intensive control of atrophic rhinitis in elite breeding and multiplier herds in Sweden. In: International pig veterinary society congress proceedings. Melbourne, Australia. 2000.

[17] Anonymous. Surveillance of infectious diseases in animals and humans in Sweden 2016, SVA reports serie 45 ISSN 1654-7098. Salmonellosis, N.V.I. (SVA), Editor. Uppsala, Sweden. 2016. p. 77–90 .

[18] EFSA. Analysis of the baseline survey on the prevalence of Salmonella in holdings with breeding pigs in the EU (2008). Part A: Salmonella prevalence estimates. EFSA J.

[19] Wallgren P, Vallgårda J (1993). Serogrisproduktion - presentation, definition och kravlista [in Swedish] Production of Specific Pathogen Free pigs - presentation, definition and list of requrements. SvenskVeterinärtidning..

[20] Xiao L, Limor K, Royer J, Lal M (2000). Identification of species and sources of *Cryptosporidium* oocysts in storm waters with a small-subunit rRNA-based diagnostic and genotyping tool. Appl Environ Microbiol..

[21] Koehler A, Korhonen P, Hall R, Young N, Wang T, Haydon S (2017). Use of a bioinformatic-assisted primer design strategy to establish a new nested PCR-based method for *Cryptosporidium*. Parasit Vectors..

[22] Hamnes I, Gjerde B, Forberg T, Robertson L (2007). Occurrence of *Cryptosporidium* and *Giardia* in suckling piglets in Norway. Vet Parasitol.

[23] Enemark H, Ahrens P, Bille-Hansen V, Heegaard P, Vigre H, Thamsborg S (2003). *Cryptosporidium parvum:* infectivity and pathogenicity of the ‘porcine’ genotype. Parasitology.

[24] Thompson R, Koh W, Clode P (2016). *Cryptosporidium*—What is it?. Food Waterborne Parasitol..

[25] Enemark H, Ahrens P, Lowery C, Thamsborg S, Enemark J, Bille-Hansen V, Lind P (2002). *Cryptosporidium andersoni* from a Danish cattle herd: identification and preliminary characterisation. Vet Parasitol.

[26] Kváč M, Němejc K, Kestřánová M, Květoňová D, Wagnerová P, Kotková M, Rost M (2014). Age related susceptibility of pigs to *Cryptosporidium scrofarum* infection. Vet Parasitol.

[27] Wang R, Qiu S, Jian F, Zhang S, Shen Y, Zhang L (2010). Prevalence and molecular identification of *Cryptosporidium* spp. in pigs in Henan, China. Parasitol Res..

[28] Zhang W, Yang F, Liu A, Wang R, Zhang L, Shen Y (2013). Prevalence and genetic characterizations of *Cryptosporidium* spp in pre-weaned and post-weaned piglets in Heilongjiang Province, China. PLoS ONE..

[29] Zhao W, Wang J, Ren G, Yang Z, Yang F, Zhang W (2018). Molecular characterizations of *Cryptosporidium* spp. and *Enterocytozoon bieneusi* in brown rats (*Rattus norvegicus*) from Heilongjiang Province, China. Parasit Vectors..

[30] Backhans A, Jacobson M, Hansson I, Lebbad M, Lambertz S, Gammelgård E (2012). Occurrence of pathogens in wild rodents caught on Swedish pig and chicken farms. Epidemiol Infect.

[31] Björkman C, von Brömssen C, Troell K, Svensson C (2018). Disinfection with hydrated lime may help manage cryptosporidiosis in calves. Vet Parasitol.

[32] Xiao L, Herd R, Bowman G (1994). Prevalence of *Cryptosporidium* and *Giardia* infections on two Ohio pig farms with different management systems. Vet Parasitol.

[33] Ryan U, Samarasinghe B, Read C, Buddle J, Robertson I, Thompson R (2003). Identification of a novel *Cryptosporidium* genotype in pigs. Appl Environ Microbiol.

[34] Xiao L, Bern C, Arrowood M, Sulaiman I, Zhou L, Kawai V (2002). Identification of the *Cryptosporidium* pig genotype in a human patient. J Infect Dis.

[35] Kvác M, Kvetonová D, Sak B, Ditrich O (2009). *Cryptosporidium* pig genotype II in immunocompetent man. Emerg Infect Dis.

